# 

**Published:** 1829-10-01

**Authors:** 


### CONTENTS

OF THE

### MEDICO-CHIRURGICAL REVIEW.

No. XXII. OCTOBER 1, 1829.

### REVIEWS.

I.

ON FEMALE DISEASES.

1. Dr. Hall's Commentaries on Female Diseases . .. .. .. .. .. .. .. } 289
2. Dr. Gooch on Diseases peculiar to Females .. .. .. .. .. .. .. .. }

II.

TRANSACTIONS OF THE MEDICAL AND PHYSICAL SOCIETY OF CALCUTTA.

1. On Diseases of the Spleen. By Mr. Twining.. .. .. .. .. .. .. .. .. 314
2. On the Medical Topography and Diseases of Arracan. By Mr. Burnard.. .. 318

III.

Mr. Madden's Travels. Plague, &c. .. .. .. .. .. .. .. .. .. .. .. .. 323

IV.

Mr. Bell on the Anatomy, Physiology, and Diseases of the Teeth.. .. .. .. 328

V.

Pathological Observations.—Part II. On Continued Fever, Ague, Tic Doloureux, Measles, Small Pox, and Dropsy, &c. By William Stoker, M.D. .. .. .. 337

VI.

1. On Disorders of the Mind in Lying-in Women. By Robert Gooch, M.D. .. 359
2. On Puerperal Mania. By Dr. Marshall Hall .. .. .. .. .. .. .. .. 363

VII.

TRANSACTIONS OF THE MEDICAL AND PHYSICAL SOCIETY OF CALCUTTA.

1. An Account of an Epidemic Malignant Ulcer, or Hospital Gangrene. By J. Adam, M.D. .. .. .. .. .. .. .. .. .. .. .. .. .. .. .. .. ... .. 371
2. On Gangrenous Ulcer. By J. Leslie, Esq. .. .. .. .. .. .. .. .. .. .. 374

VIII.

MEDICO-CHIRURGICAL TRANSACTIONS.

Contribution to the Pathology of Phlegmatia Dolens. By Robert Lee, M.D. &c. 380

IX.

MEDICO-CHIRURGICAL TRANSACTIONS.

Observations on the Local Diseases termed Malignant; Part I. By Benjamin Travers, F.R.S. &c. &c. .. .. .. .. .. .. .. .. .. .. .. .. .. .. .. 385

X.

On the Irritable Uterus. By Robert Gooch M.D. &c. Art. IV. .. .. .. .. .. 404

XI.

On Diseases of the Teeth. By Thomas Bell, F.R.S. &c. Art. II. .. .. .. 408

XII.

Elements of Medical Statistics. By F. Bisset Hawkins, M.D. &c. &c... .. .. 420

### PERISCOPE.

Dr. Darwall on Cerebral and Spinal Irritation .. .. .. .. .. .. .. .. .. .. 433
Carditis Simulating Chorea .. .. .. .. .. .. .. .. .. .. .. .. .. .. .. 439
Subcarbonate of Iron in Tetanus .. .. .. .. .. .. .. .. .. .. .. .. .. .. 440
M. Roux on Fistula of the Parotid Duct .. .. .. .. .. .. .. .. .. .. .. 443
Mr. Brodie on Aneurisms by Anastomosis .. .. .. .. .. .. .. .. .. .. .. 444
Ulceration of the Auricle of the Heart .. .. .. .. .. .. .. .. .. .. .. .. 446
On the Effects of Biliary Calculi .. .. .. .. .. .. .. .. .. .. .. .. .. 447
Fracture of Bones from trifling violence in Cases of Cancer .. .. .. .. .. .. 449
Some Observations on Chorea.. .. .. .. .. .. .. .. .. .. .. .. .. .. .. 481
Peculiar Odorous Principle in the Blood of Man. By M. Barruel .. .. .. .. .. 483
Mr. Battley's Liquor Cinchonæ Cordifoliæ .. .. .. .. .. .. .. .. .. .. .. 484
Two remarkable Cases of Intermittent Aphonia. By Messrs. Rennes and Ollivier.. 488
On Spasm or Cramp of the Stomach. By Dr. Macfarlane .. .. .. .. .. .. 489
Elephantiasis Græcorum and Elephantiasis Arabum .. .. .. .. .. .. .. .. 492
Employment of Tartrite of Antimony externally. By Dr. Fontaneilles .. .. .. 495
Case of Perforation of the Stomach .. .. .. .. .. .. .. .. .. .. .. .. .. 496
Mr. Ceely on the Nitrate of Silver.. .. .. .. .. .. .. .. .. .. .. .. .. .. 496
A Letter to John Bull on the Dissection of his Body. By Gracchus .. .. .. 529
Memoir on Softening of the Stomach in Infants. By Dr. Fels of Leipsig .. .. 530
Adipocire in the Fluid of a Hydrocele .. .. .. .. .. .. .. .. .. .. .. .. .. 533
Mr. Brodie and Dr. Hastings on Cysts connected with the Convex Surface of the Liver .. .. .. .. .. .. .. .. .. .. .. .. .. .. .. .. .. 535
Some Cases of Neuralgia. By Dr. Passagay .. .. .. .. .. .. .. .. .. .. 538
On Otorrhœa Purulenta; with Cases. By Dr. Burne .. .. .. .. .. .. .. .. 538
New Mode of stopping Hæmorrhage by twisting the Mouths of the Vessels. By M. Amussat .. .. .. .. .. .. .. .. .. .. .. .. .. .. .. .. .. 545
Mr. Stephens' Views on the Subject of Hernia; Cases and Remarks .. .. .. 546
Poisoning from Medicine prescribed by Mistake .. .. .. .. .. .. .. .. .. 551
Regulations of the Edinburgh College of Surgeons, respecting the Qualifications of Candidates .. .. .. .. .. .. .. .. .. .. .. .. .. .. .. .. .. .. 552

### CLINICAL REVIEW.

### Gloucester Infirmary.

1. Dr. Baron on Iodine .. .. .. .. .. .. .. .. .. .. .. .. .. .. .. .. .. 451
2. A. Tumour growing from the Dura Mater at the Basis Cranii .. .. .. .. .. 512
B. Cataracts successfully treated by couching .. .. .. .. .. .. .. .. .. 513

### Hôpital St. Louis.

1. Lectures on Diseases of the Skin;—Acne—Lupus—Porrigo—Psoriasis—Scabies .. .. .. .. .. .. .. .. .. .. .. .. .. .. .. .. .. .. 453
2. A. Sycosis Menti.. .. .. .. .. .. .. .. .. .. .. .. .. .. .. .. .. .. 502
B. Lupus—Frictions with the Deuto-Ioduret of Mercury .. .. .. .. .. .. 504
C. Erythema Circinnatum .. .. .. .. .. .. .. .. .. .. .. .. .. .. .. 504
D. Rubeola—bad Effects of Blood-letting .. .. .. .. .. .. .. .. .. .. 504
3. On Diseases of the Skin
A. On Lichen Agrius.. .. .. .. .. .. .. .. .. .. .. .. .. .. .. .. .. 567
B. On Lichen Gyratus and Lichen Circumscriptus .. .. .. .. .. .. .. 568

C. On Herpes .. 568

D. On Eczema .. 570

E. On Psoriasis .. 571

F. On Elephantiasis Arabum .. 571

ST. GEORGE'S HOSPITAL.

1. Sloughy Abscesses—Ulceration of Cartilages .. 456
2. State of the Heart and Arteries in Apoplexy .. 458
3. Ulcerated Nævus .. 459
4. Diseases of the Heart
A. Dilatation with Hypertrophy of Left Ventricle—Pus in the Polypi .. 518
B. Peripneumony—Dilatation of the Heart .. 521
5. Diseases of the Heart .. 556
A. Hypertrophy with little Dilatation of Left Ventricle .. 557
B. Hypertrophy with some Dilatation of Left Ventricle—Enlargement of the Liver .. 558
C. Adhesions of the Pericardium—Hypertrophy and Dilatation of Left Ventricle .. 559
D. Hypertrophy of Left Ventricle—Adhesions of the Pericardium? .. 560
E. Great Dilatation and Hypertrophy of the Heart—curious Disorganization of the Liver .. 561

LA CHARITE.

1. Diseases of the Prostate Gland and Vesiculæ Seminales .. 459
2. Pleuritis Hæmorrhagica—Operation .. 462

ST. THOMAS'S HOSPITAL.

1. Venereal Sore of the Mamma, from Secondary Syphilis in a Child .. 464
2. Curious Case of Phlebitis .. 465

HÔPITAL ST. ANTOINE.

1. Remarkable Case of Masked Ague .. 465

EDINBURGH ROYAL INFIRMARY.

1. Enormous Hypertrophy of the Heart .. 466

2. Laryngitis—successful Tracheotomy .. 467

3. A. Temporal Aneurism from Arteriotomy .. 533

B. Disease and Removal of the Bones of the Cranium .. 534

WINCHESTER COUNTY HOSPITAL.

1. Ununited Fracture cured by Seton .. 468

HÔTEL DIEU.

1. Ligature of the Artery beyond the Aneurismal Tumour, with the Results and Criticisms .. 469

2. A. Successful Extirpation of the Uterus .. 564

B. Supposed Foreign Body in the Ear—Hydrocephalus, &c. .. 565

C. Operation for an excessive Prolongation of the Mucous Membrane of the Upper Lip .. 566

D. M. Dupuytren on Hernia of the Muscles .. 566

WESTMINSTER HOSPITAL.

1. Case of violent and long-continued Head-ache removed by Erysipelas on the Face .. 473

ROYAL WESTMINSTER OPHTHALMIC HOSPITAL.

1. Operations for Cataract .. 474

2. Mr. Guthrie on the Oleum Terebinthinæ in Inflammation of the Iris and Choroid Coat .. 525

HÔPITAL DES ENFANS.

1. Collections of Pus in the Cavities of the Heart .. 475

WORCESTER INFIRMARY.

1. On the Use of the Stethoscope in Pulmonary Affections. By Chas. Hastings, M.D. .. 497

GLASGOW ROYAL INFIRMARY.

1. A. Disease of the Maxillary Sinus .. 505
   B. Affections of the Eye .. 506
   C. Lithotomy after the Operation for Fistula in Ano .. 508
   D. Fractures and Amputations .. 508

MADRAS MILITARY HOSPITAL.

1. Remarks on Hypochondriasis, Melancholia, and Insanity. By J. Annesley, Esq. 510

BRISTOL INFIRMARY.

1. Cases of Aneurism
   A. Rupture of Popliteal Aneurism—Amputation.. .. 513
   B. Spontaneous Cure of Femoral Aneurism—Gangrene .. 515
   C. External Iliac Aneurism—Bronchitis—other Diseases—Operation—Death. Remarks.. .. 516

HOSPICE DE LA SALPETRIERE.

1. Paralysis, Colic, &c. connected with Intestinal Worms .. 563

MAISON ROYALE DE SANTE.

1. Madame Boivin on Polypus of the Uterus and Pregnancy.. .. 571

MISCELLANIES.

1. Remarks on a Class of Pseudo-inflammatory Affections. By Mr. Newstead.. 478
2. Mr. Davis on Ergot of Rye .. 480
3. Mr. R. Alcock's Communication respecting Baron Heurteloup's instruments.. 573

Bibliographical Record .. 575

